# Exploration of the Feasibility of Remote Assessment of Functioning in Children and Adolescents with Developmental Disabilities: Parents’ Perspectives and Related Contextual Factors

**DOI:** 10.3390/ijerph192215101

**Published:** 2022-11-16

**Authors:** Beatriz Helena Brugnaro, Fabiana Nascimento Vieira, Gesica Fernandes, Olaf Kraus de Camargo, Laís Fumincelli, Ana Carolina de Campos, Silvia Letícia Pavão, Nelci Adriana Cicuto Ferreira Rocha

**Affiliations:** 1 Child Development Analysis Laboratory (LADI), Department of Physical Therapy, Federal University of São Carlos (UFSCar), São Carlos 13565-905, SP, Brazil; 2 CanChild, Department of Pediatrics, McMaster University, Hamilton, ON L8S 1C7, Canada; 3 Department of Nursing, Federal University of São Carlos (UFSCar), São Carlos 13565-905, SP, Brazil; 4 Department of Prevention and Rehabilitation in Physical Therapy, Federal University of Paraná, Curitiba 80060-000, PR, Brazil

**Keywords:** feasibility, assessment, remote, pandemic, child, family, disability

## Abstract

The COVID-19 pandemic interrupted face-to-face health services, leveraging telehealth strategies. The aim of this cross-sectional study was to investigate, from a parent’s perspective, the feasibility of a remote assessment of functioning in children with developmental disabilities during the pandemic and related contextual factors, based on how parents carry out the assessment. Parents of children with developmental disabilities (mean age = 7.56 ± 3.68) responded to a remote assessment via electronic forms and telephone interview. We analyzed parents’ perspectives about the feasibility of the assessment. We also tested the association between feasibility score and sociodemographics/pandemic experience. Regression analysis tested if children’s functioning characteristics predicted feasibility. A total of 57 mothers completed the remote assessment, and more than 95% did not report difficulties in accessing/responding to electronic forms. They scored remote assessment as easy and feasible, and reported no difficulties with telephone interview. Greater feasibility rates were related to lower maternal age (rho Spearman = −0.290; *p* = 0.029). The model shows that children’s characteristics predicted 20.4% of feasibility (*p* < 0.005). Remote assessment showed to be feasible. Younger mothers might consider easier-to-use technologies, beyond considering remote assessment more viable. These results can guide the next steps in research and remote clinical practice.

## 1. Introduction

Children with developmental disabilities present complex health needs and multiple potentialities, which require therapeutic approaches using the International Classification of Functioning, Disabilities and Health (ICF) biopsychosocial model of health as a conceptual outline. This approach goes beyond consideration of the impairments of body structure and functions, to recognize and promote functioning [1,2].

The relevance of the biopsychosocial model of health for families who have children with developmental disabilities significantly increased in the context of the COVID-19 pandemic. This is because social distancing has brought with it extensive changes in the lives of families, with particular potential impact on many domains of functioning at the same time [3]. The protective measures undertaken, such as social distancing and interruption of face-to-face activities [4,5,6,7,8], more than preventing the spread of the COVID-19, interrupted a series of therapies and other activities to promote the development of these children. Thereafter, countless families with children and adolescents presenting developmental disability were left out by the impossibility of continuing therapeutic care or face-to-face guidance for follow up [5,9].

Accordingly, telehealth strategies and remote assessment gained prominence, allowing continuity of professional–family contact, thus ensuring assessment and monitoring of functioning, even in a period of physical distancing [6,10,11]. Remote health care involves both patients’ evaluation and intervention. Nevertheless, the implementation of remote care programs occurred despite the lack of specific technical directions addressing physical security of the patients, the confidentiality of their data, and ethical aspects involving virtual data management and other investigations about the feasibility of the processes used in remote assessment [4,5,9,12,13]. In this way, for telehealth strategies for children with disabilities to be well targeted and reliable, it is important that remote assessments are effective, and parents feel comfortable with this kind of approach. A better exploration of the issues related to the remote assessment, especially conducted under the parents’ perceptions and opinions, might better guide these services, improving quality of telehealth, thus allowing its use as an alternative therapeutic care [14,15,16].

Remote assessment reduces barriers and allows broad access to monitoring the development of children, expending lesser financial resources [4,5,11,12,17,18]. However, it is necessary to first investigate the feasibility of a remote assessment of children’s health conditions under the light of the biopsychosocial model, considering parents’ perspectives of this evaluation, then ensuring family-centered care and direct involvement of parents in care-taking, listening to their needs, and establishing a partnership between family and therapists [10].

Contextual factors may be associated with the parents’ perception of the feasibility of remote assessment, as the interaction with the assessment may vary depending on the family’s socioeconomic level, maternal age, mother’s type of work and the experience of social distancing, considering the time and the type lived in social distancing, as well as whether the child continues to undergo therapy during social distancing. These factors can be associated and influence adherence to remote assessment. It is therefore relevant to investigate these aspects to better understand the parents’ perception of feasibility under the COVID-19 context.

Thus, in the present study, we aimed to investigate, from parents’ perspectives, the feasibility of using a remote assessment of functioning, for children and adolescents with developmental disabilities, using the biopsychosocial approach as a conceptual outline, through electronic forms and telephone interview directed to the parents during the COVID-19 pandemic. Additionally, we aimed to explore if contextual factors are related to perceived feasibility.

This study is part of a larger study during the COVID-19 pandemic, which justifies the choice of the instruments used. This larger study aimed to follow up remotely some aspects of functioning of children with developmental disabilities during the pandemic period. Therefore, the research question of the larger study guided the selection of measures. We expected to find a good acceptance of the remote assessment of functioning by parents. Our hypothesis was based on the logistical ease of a remote assessment, and because parents can see on remote assessment the possibility of maintaining health care, even during social distancing. We believed that—due to the impossibility of a personal face-to-face contact—remote assessment with a biopsychosocial approach would be recommended as an alternative, in addition to reducing the costs of the assessment process.

## 2. Methods

### 2.1. Participants

A non-probabilistic sample was used for convenience. We invited parents of children and adolescents with developmental disabilities through university and rehabilitation institutions social networks and communication media (e mail). We evaluated parents of children and adolescents—from 3 to 17 years old—with diagnosed developmental disabilities. Therefore, we included children with physical, intellectual, or behavioral disabilities, such as cerebral palsy, autism, Down syndrome or myelomeningocele. The inclusion criteria were as follows: parents who signed the electronic informed consent and children who provided informed assent. The exclusion criteria were: children with a non-formal diagnose as listed above, whose parents did not complete all assessments for any reasons or participants did not sign the consent/assent forms. We designated age groups based on the instruments the Young Children’s Participation and Environment Measure (0–5 years) and the Participation and Environment Measure for Children and Youth (6 to 17 years), which assess the participation of the child/adolescent at home [19,20]. The sample size was calculated a priori, considering the effect size = 0.35, α = 0.05; power = 0.90, and number of predictors = 6. The required sample size was 57 participants.

### 2.2. Measuring Instruments

#### 2.2.1. Questionnaire on the Feasibility of the Research According to the Parents’ Perspective—By Online Surveying

Researchers designed an online standardized questionnaire (electronic form) about the parents’ opinions on the online survey, with an average time to complete of 10 min. The questionnaire addresses aspects regarding the level of difficulty in answering the questions, the length of the questionnaire, whether they would recommend other people to participate in the survey, and any suggestions about the assessment or the online survey/telephone interview. The complete form can be found in supplementary material 1 (Table S1), with questions regarding the feasibility of the assessment performed through electronic forms and telephone interview. The answers of the 7 (seven) questions were converted into a score scale for each question (called *feasibility score*). The final score was obtained by the sum of all the answers (including the questions regarding electronic forms and telephone interview together) with higher scores indicating higher feasibility.

This questionnaire was applied after the participants finished the assessment, as a way to obtain the participants’ opinions on the feasibility of remote assessment of functioning according to their perspective.

#### 2.2.2. COVID-19 Questionnaire: Sociodemographic Data and Pandemic Experience—By Online Surveying

A standardized questionnaire with sociodemographic data including maternal age and schooling, socioeconomic classification (Brazilian Association of Research Companies, ABEP), housing characteristics, type and duration of social distancing experienced by the mother and child, maternal employment situation during the pandemic, and work regimen (in-person, remote or not working). This survey had an average time to complete of 10 min, under an electronic form. All data involving the pandemic were collected as a way of understanding the child’s family context at the time of assessment. Moreover, the data are described in the section dedicated to evaluating environmental factors.

#### 2.2.3. Functioning Assessment—Mixed by Online Surveying and by Telephone Interview

**Figure 1:**
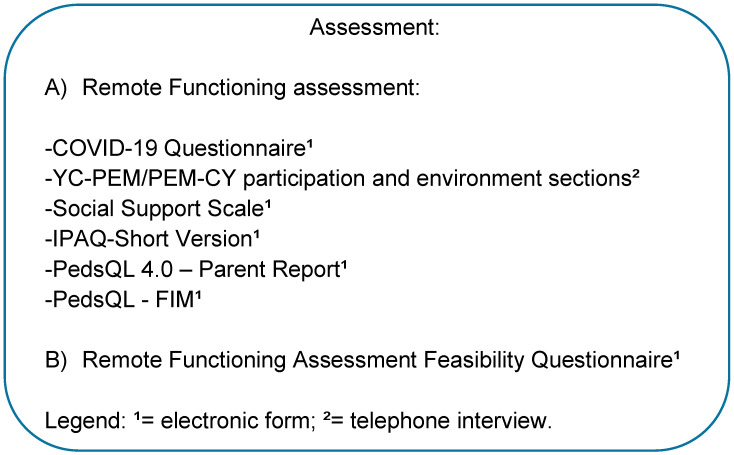
Assessment performed remotely.

The assessment of functioning including the components of the ICF domain is highlighted in Figure 1, which illustrates the instruments described below.

The Participation and Environment Measure for Young Children’s (YC-PEM) and for Children and Youth (PEM-CY)—participation part and environment part for home section—were the only measures applied by telephone interview with a duration of 30 min. All interviews were conducted by the same researcher, trained, in order to minimize bias. These measures are correspondents that informed about the participation at home of the child and the environmental facilitators and barriers for home participation, according to child’s age (YC-PEM: 0–5 years; PEM-CY: 6–17 years). YC-PEM/PEM-CY were intended to be applied by paper. Other measures were all collected by online surveying (via electronic form). To assess environmental factors, we used the Social Support Scale (average of 7 min to complete), and the characteristics maternal age and schooling, characteristics of housing, socioeconomic classification (ABEP), and characteristics of social distancing, in the socio demographic form. Additionally, we collected the Body Mass Index (BMI) as one aspect of body functions and structures, and the diagnose, age and sex of children for personal factors (average 5 min to complete). The International Physical Activity Questionnaire short version (IPAQ-short version) was also applied via electronic form (average of 7 min to complete), to inform aspects related to activities, and was intended to be applied by paper. In addition, we also applied via electronic forms the Pediatric Quality of Life Inventory (PedsQL) version 4.0 (average of 7 min to complete), and the Family Impact Module (PedsQL-FIM) (average of 7 min to complete), that were intended to be applied by paper. All instruments are described in supplementary material 2 (Table S2).

### 2.3. Procedures

This was a cross-sectional and exploratory study that followed the recommendations of the Checklist for Reporting Results of Internet E-Surveys (CHERRIES) statement guideline, considering the research application in Brazil and country-specific standards, and the Strengthening the Reporting of Observational Studies in Epidemiology (STROBE) checklist. This study followed resolutions 466/2012 and 510/2016 of the National Health Council and was approved by the local ethics committee (CAAE: 31786920.8.1001.5504).

The assessments occurred between May and August 2020, in Brazil, and were remote surveys.

**Figure 2:**
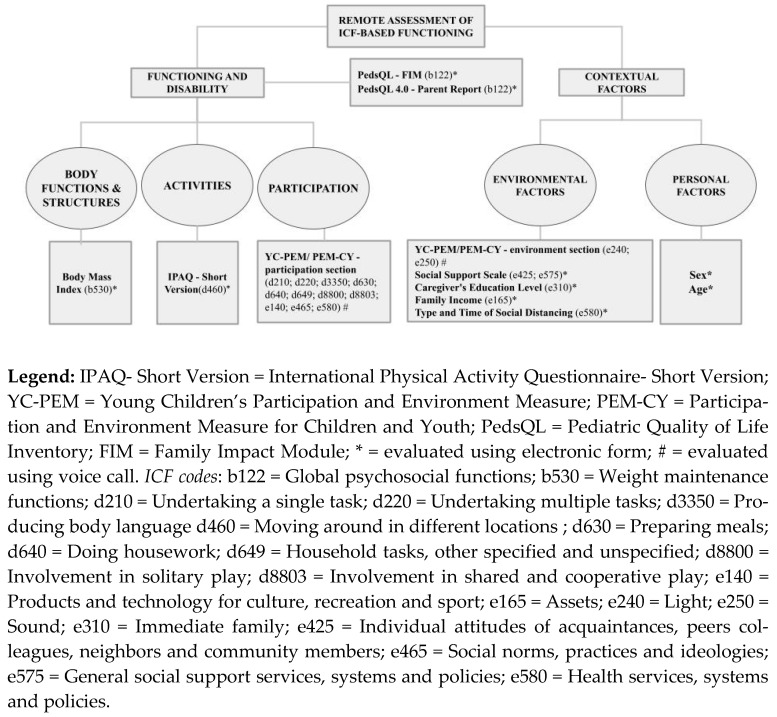
Assessment of functioning.

The assessment of the functioning of children and adolescents with developmental disabilities involved aspects of each health domains of the ICF—body structure and functions, activities, participation, environmental factors, and personal factors—as described in Figure 2. Participants were told to complete each assessment within 10 calendar days. We sent reminders every 2 days to participants, remembering them to answer the questions. There was no randomization of the items of the questions since the assessment followed standardized instruments with a fixed structure.

## 3. Data Analysis and Statistical Tests

The feasibility data of the research were categorically described and analyzed. We calculated total scores and the frequency of occurrence percentages. Data analysis was performed using Excel ^®^. Spearman’s correlation analysis tested associations between the feasibility score (obtained by sum of each categorized score) of remote assessment of functioning and the family contextual factors. The correlations were classified according to Cohen and Holliday’s classification (1982) (up to 0.19: very weak; between 0.20 and 0.39: weak; from 0.40 to 0.69: moderate; from 0.70 to 0.89: strong; and from 0.90 to 1: very strong).

Furthermore, multiple linear regression analyses using the enter method were performed to verify whether contextual factors (family socioeconomic class, maternal age, type of maternal social distancing (total, partial or non-distancing), duration of social distancing (up to 1 month, 1 to 2 months, 2 to 3 months, 3 to 4 months, more than 4 months), whether or not the child received in-person therapy during the pandemic and the mother’s work regimen (does not work, works remotely or works in-person)) are associated with parents’ perceived viability in the remote assessment of functioning. For the statistical analysis of correlation and linear regression, we summed up all the points obtained in each item of the feasibility questionnaire score, in a range of 0–12. Greater scores indicated greater feasibility. Missing data were treated as a missing.

## 4. Results

**Figure 3:**
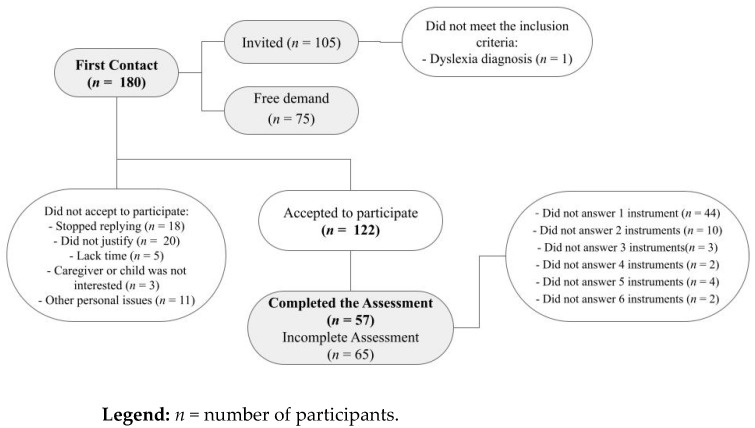
Flowchart of the participants.

Although the survey invited parents (mothers or fathers), all participants were mothers. The researchers initially approached 180 mothers. Of these, 122 accepted to participate, and 57 fully completed the assessment. Figure 3 illustrates the flowchart of participants recruitment, with the reasons for the drop-out throughout the research. Table 1 illustrates the sociodemographic data and some characteristics of the parents’ experience during the pandemic. All participants were able to read and complete the questionnaires. Table 2 shows the frequency of feasibility answers and the feasibility total score. The response time of the assessment was: 35 participants (61.4%) finished within 10 days, and 22 participants (38.6%) finished late, with an average time to finish of 14.8 days for all participants.

Correlation analyzes showed that lower maternal age was correlated with greater feasibility of remote assessment (rho Spearman = −0.290; *p* = 0.029, weak correlation). The complete results of the correlation can be found in supplementary material 3 (Table S3). The multiple linear regression model is represented in Table 3.

## 5. Discussion

### 5.1. Data on the Feasibility of Remote Assessment from the Parent’s Perspective

For the assessment by electronic forms, the results confirmed the hypothesis that parents consider remote assessment of functioning feasible and even more viable than face to face, supporting the validity of the obtained results.

In the questionnaire related to the telephone interview, the majority of mothers reported no difficulties for answering the survey. When asked which modality of evaluation they preferred for assessment, online or face to face, most participants preferred electronic forms. Only 10.5% reported preferring a face-to-face assessment, indicating that the majority of participants were satisfied with the remote assessment.

Indeed, our results are in line with previous studies comparing paper versus web-based modalities [21], paper versus tablet [22], and paper versus application [23]. Advances in technology over the past two decades have led clinicians to reconsider the way clinical care is administered and research is conducted [24]. The use and feasibility of electronic forms for patients’ assessment are not a recent issue in health field [21,22,25], although it has largely increased during the pandemic. In 2010, Touvier et al. found that web-based questionnaires provide information of equal or superior quality when compared to the paper-based method. In a systematic review published by Meirte et al. (2020) [26], the authors concluded that electronic assessments are feasible and accepted not only in health professionals, but also in patients with different health conditions.

Most of the studies addressing the viability and feasibility of remote assessments are directed to adult and elderly people [26]. Before the pandemic we only found the study of Raat et al. (2007) [25] who supports the feasibility, internal consistency reliability of remote assessments for quality of life in children. After 2020, the social distancing imposed by COVID-19, the number of studies addressing these issues for children and adolescents increased, presenting positive results and good perspectives for remote assessment [24,27,28].

According to Montes et al. (2022) [24], who provided a roadmap for post-pandemic remote assessment, surveys and patient reported outcomes show higher feasibility and require less effort to undertake in remote settings. This kind of assessment may be easier to complete remotely. Nevertheless, whilst remote motor function assessments require additional investigation to be established as reliable and valid, advantages such as speed and convenience of being at home are consistent. Anyway, despite the need of further research and potential validations, the advantages are unanimous [24,27,28]. Beyond the fact that almost everyone owns smartphones, lightweight computers or tablets [26], the use of online surveys allows clinicians and researchers to access wide and diversified populations and achieve quick return [21], which could potentially increase the diversity and representativeness of study samples. Finally, these methods have significant potential to facilitate the harmonization. Cost reductions is another important aspect raised by remote assessments, since they do not require travels, beyond being less burdensome for participants [28].

Considering the advantages based on the point of view of children and their families, the use of technology for assessment and rehabilitation might open opportunities for children with developmental disabilities and their families to be more involved in the planning, implementation and evaluation of their care [23]. Moreover, by reducing barriers between therapist and patient, remote assessments allow a stronger structuring for implantation of family centered therapy [3].

Lastly, we believe that the convenience of answering electronic forms, with no need to schedule an appointment or need to be ready and organized to leave the house, explains why it is the preferred modality of the respondents. This might have had an especial importance during the pandemic, in which domestic routine were being adjusted by parents to adapt to the new formats of remote school [29] and work [30].

We highlight that despite the advantages of the remote assessment regarding the freedom to respond at any time, the average number of days (14.8) to finish the assessment was higher than desired, even with reminders being sent every 2 days In line with our results, a literature review by Meirte et al. (2020) [26] found that research participants prefer technological modalities when compared to paper-based modalities, in addition to considering them easier, faster, and more cost-effective. They also highlighted that the original instruments, when adapted for electronic forms, do not present results or scores as the paper-based versions [26]. This reinforces the importance of new studies to verify the validity and reliability of remote assessments of the instruments in comparison to face-to-face assessment.

### 5.2. Associations between the Feasibility of Remote Assessment and the Contextual Factors

Testing the association between the feasibility of remote assessment and contextual factors of the children’s families, we only found significant relationships for mothers’ age. Indeed, higher feasibility rates reported by the mothers were associated with lower maternal age. This result confirms the hypothesis initially drawn. Younger mothers are more familiarized with technologies since they were born in a period in which there is greater access to technological resources [31], which may explain why they have found the research to be more feasible.

Other studies suggested satisfactory levels regarding the preference for electronic forms for younger participants with higher levels of education, stable employment, and residence in the city (urban area) [26,32]. In fact, people with previous experience in electronic forms—when compared to people with experience only in paper-based assessments—are more interested in using electronic forms [33]. Meirte et al. (2020) have cited in their systematic review the influence of age on feasibility and time to respond the assessment, impacting their experience with online versions. As for the older participants, a study showed that 49% of the older participants needed help from a family member or researcher to complete the online questionnaire, due to lack of familiarity with the computer or difficulties using the mouse [34]. Usually, older people have reservations concerning modern computer technology and need to be properly approached. Otherwise, Salaffi et al. (2013) did not find an association between the age of the respondents and aspects of the feasibility of remote assessment. Nevertheless, some strategies might be adopted by the researchers and clinicians aiming to avoid negative aspects of age when using remote evaluation for children such as educational sessions on the use of the digital app and ensuring sufficient support for families [26].

We point out that although maternal age was associated with feasibility rates of remote evaluation, the significant correlation we found was weak, that is, the results could be modified considering the characteristics of the sample. Possibly, there may not have been enough variability in the feasibility ratings, considering the small standard deviation we found. So, in general, we need to be cautious interpreting the results, and further investigations are needed.

Together, the addressed families’ contextual factors explained 20.4% of the feasibility rates for remote assessment of functioning. The addressed contextual factors of the families which entered in the regression model were maternal age, socioeconomic level, maternal type of social distancing, maternal time of social distancing, type of maternal work and maintenance of in person therapy during the pandemic. Although the only individual contextual factor associated with feasibility rates has been maternal age, the set of variables that entered in the regression model, which include maternal age variable, were found to be determinants of the feasibility rates of remote assessment. Potentially, characteristics of type and time of adopted social distancing and the type of maternal work, together with maternal age, seems to impact the acceptance and viability of using remote assessments by the mothers. This prediction relationship seems even more relevant in the pandemic scenario, since mothers all around the world had to adapt to carry out work and educational activities remotely, and thus, their job characteristics, socioeconomic level influenced their judgments about remote assessment.

Accordingly, based on these results, socioeconomic data from the families, time availability of the mothers, schooling level and even maternal age should be considered during study designs which aim use remote assessment of children’s functioning, thus ensuring better adherence of families to the evaluation process.

Therefore, further studies are still needed to exactly define how socioeconomic factors can influence remote assessment of functioning, and to propose possible strategies for further investigation and adaptation to this group, facilitating its use and, therefore, the remote assessment of functioning. In addition, it is worth mentioning that the upper classes may show more interest in the research [35], probably because they understand the importance of research to the formation of knowledge, thus favoring their participation in this study.

## 6. Conclusions

The remote assessment of functioning using the instruments selected for this study, which included electronic forms and telephone interview, proved to be feasible and without difficulties for the outcome measures we used. For contextual factors, younger mothers showed greater ease in participating, probably because they were more familiar with digital tools. Thus, aspects that motivate and facilitate the use of remote assessment of functioning of children/adolescents with developmental disabilities should be encouraged, as they can be great allies to foster professional–family contact virtually. By better understanding these aspects, it is possible to perform remote assessments during and after the pandemic, favoring the development of this target population and reinforcing family-centered care.

## 7. Clinical Implications

Remote assessments, which gained prominence during the COVID-19 pandemic, represent advances in telehealth, as they enable the maintenance of contact between health professionals and populations with health care needs. We highlight the importance of listening to parents about this kind of evaluation, trying to understand their points to improve parental adherence to assessments. In addition, remote assessments may diminish financial, social, or locomotion issues. Thus, the pandemic provided an opportunity to advance remote care, and this study illustrates the feasibility of a remote functioning assessment, from the perspective of parents. We believe that this assessment model will be maintained even after the pandemic, and thus studies that analyze its feasibility are increasingly necessary to standardize and guide professionals on how to evaluate by telehealth, prioritizing the biopsychosocial model recommended by the ICF. Therefore, this is an exploratory study that helps guide the next steps in research and remote clinical practice.

## 8. Study Limitations

The sample was quite heterogeneous, which hinders the internal validity of the results, but reinforces the external validity to the overall population of children with disabilities, in its multiplicity and variety of clinical health conditions. In addition, the assessment occurred entirely at a distance, without validation of the assessment by electronic forms and telephone interview. In this sense, the researchers tried to explain in the introductory text of the forms how the participants should respond, and they were available to answer questions. For the telephone interview, all assessments, without exception, were performed by the same researcher, in order to maintain the reliability of the assessment and reduce bias. We also point out that families without access to these resources probably cannot be contemplated by the results.

## 9. Future Studies

Based on the results obtained in this study, we suggest that further research should verify the validity and reliability of the remote assessment of the instruments used through psychometric test analyses—for example, Vineland-3 by Pearson [36]. In addition, we recommend that new ways of explaining the instruments be tested to ensure that respondents understand the questions and answer options, in a standardized way, aiming to maintain test–retest and inter-rater reliability. Thus, future studies will ensure the validity and reliability of instruments of the remote assessment of functioning, which was considered a viable option by the participants of this study, including longitudinal assessments, to verify the adherence of mothers in multiple assessments.

## Figures and Tables

**Table 1:** Sociodemographic data.

**Child’s age**	***n* = 57**
Mean	7.56
Standard deviation	3.68
**Child’ type of developmental disability**	***n* (%)**
Down syndrome	25 (43.9%)
Autism	11 (19.3%)
Cerebral palsy	10 (17.5%)
Others	11 (19.3%)
**Socioeconomic Level—ABEP**	***n* (%)**
A	5 (8.8%)
B1	4 (7.0%)
B2	16 (28.1%)
C1	16 (28.1%)
C2	13 (22.8%)
D-E	3 (5.3%)
**House characteristics**	
Type of residence:	***n* (%)**
House	42 (73,7%)
Apartment	15 (26.3%)
Number of rooms (average)	5.95
Number of residents per room (average)	0.63
**Maternal age**	
Mean	39.37
Standard deviation	7.97
Range	18–53
**Maternal schooling**	***n* (%)**
Incomplete primary education	10
Complete primary education	1
Incomplete high school	2
Complete high school	15
Incomplete higher education	1
Complete higher education	24
**Type of social distancing (child)**	***n* (%)**
Total	12 (21.1%)
Partial	43 (75.4%)
Was not in distancing	2 (3.5%)
**Type of social distancing (Mother)**	***n* (%)**
Total	4 (7.0%)
Partial	47 (82.5%)
Was not in distancing	6 (10.5%)
**Social distancing duration (Mother)**	***n* (%)**
Was not in distancing	5 (8.8%)
up to 1 month	2 (3.5%)
1 to 2 months	2 (3.5%)
2 to 3 months	15 (26.3%)
3 to 4 months	0 (0.0%)
More than 4 months	33 (57.9%)
**Does mother work before the pandemic?**	***n* (%)**
Yes	28 (49.1%)
Was not working	29 (50.9%)
**Was working during the pandemic?**	***n* (%)**
Yes, at work	11 (19.3%)
Yes, at home office	20 (35.1%)
Was not working	26 (45.6%)
**Was the child undergoing face-to-face therapy during the pandemic?**	***n* (%)**
Yes	34 (59.6%)
Not	23 (40.4%)

**Legend**: *n* = number of participants; ABEP = Brazilian Association of Research Companies.

**Table 2:** Answers obtained in the feasibility form (*n* = 57).

Questions about Electronic Forms	Possibilities of Answers	Frequency *n* (%)
Did you have any difficulty accessing or answering the form?	Yes	3 (5.3%)
	No	54 (94.7%)
If yes, why?	Problems on my internet	0 (0%)
	Difficult questions	2 (66.7%)
	Crashed the system	1 (33.3%)
	Other	0 (0%)
As for the size of the document: Do you consider that the online question script was:	Good size	49 (86.0%)
	Too long but necessary	7 (12.3%)
	Too long and unnecessary	1 (1.8%)
	Should have more questions	0 (0%)
How do you rate the ease of understanding the questions:	Very easy	9 (15.8%)
	Easy	36 (63.2%)
	Reasonable	12 (21.1%)
	Difficult	0 (0%)
The online tool is:	Feasible—can be done	57 (100%)
	Impracticable	0 (0%)
Do you recommend for other families to participate in the survey:	Yes	56 (98.2%)
	No	0 (0%)
	Maybe	1 (1.8%)
Would you like to suggest any changes to the forms?	Yes	3 (5.3%)
	No	54 (94.7%)
Questions about the Telephone Interview	Possibilities of answers	Frequency *n* (%)
Did you have any difficulties?	Yes	4 (7.0%)
	No	53 (93.0%)
If yes, why?	Very long interview	1 (25%)
	I have no time	0 (0%)
	Difficulty understanding the questions	0 (0%)
	Difficulty remembering answer possibilities	2 (50%)
	Other	1 (25%)
Which form of assessment do you consider the best?	By online form	22 (38.6%)
	By phone call	8 (14.0%)
	I see no difference between them	20 (35.1%)
	Face-to-face it would be better	6 (10.5%)
	By video recording	1 (1.8%)
Measures obtained for the feasibility total score (*n* = 57)		
**Feasibility total score**		
Mean	10.44	
Standard deviation	1.37	
Minimum–maximum score obtained	6–12	
Instrument score variation	0–12	
Percentage of mean score achieved in relation to the maximum possible	87%	

**Legend:** EF = electronic form; *n* = number of participants.

**Table 3:** Regression between contextual factor variables and remote assessment feasibility.

Outcome: Feasibility						
Predictors	B	*p*	*t* Statistics	Standardized Coefficients Beta	R^2^ Model	F
Economic Level—ABEP	0.167	0.229	1.218	0.158	0.204 *	2.131
Maternal Age	−0.044	0.051	−1.995	−0.255		
Maternal Type of Social Distancing	−0.228	0.631	−0.483	−0.070		
Maternal Social Distancing Time	0.172	0.174	1.378	0.206		
Face-to-Face Therapy during the Pandemic	0.538	0.149	1.467	0.194		
Type of Maternal Work	−0.472	0.062	−1.909	−0.264		

**Legend:** ABEP = Brazilian Association of Research Companies; *n* = number of participants; * = *p* < 0.05.

## Data Availability

The dataset supporting the conclusion of this article is available from the authors upon reasonable request.

## Supplementary Materials

The following supporting information can be downloaded at: https://www.mdpi.com/article/10.3390/ijerph192215101/s1, Table S1: Questions of the form available online—feasibility; Table S2: Description of all instruments used; Table S3: Spearman’s correlation between remote survey feasibility and contextual factor variables.
