# Supplementary material for: Exploration of the Feasibility of Remote Assessment of Functioning in Children and Adolescents with Developmental Disabilities: Parents’ Perspectives and Related Contextual Factors
Source: Int J Environ Res Public Health. 2022 Nov 16;19(22):15101. doi: 10.3390/ijerph192215101 (PMC9690884; doi:10.3390/ijerph192215101)
Supplement: Supplementary file 1 [file ijerph-19-15101-s001.zip › ijerph-1972270-supplementary.pdf]

Table S1. Questions about the feasibility regarding electronic form and telephone interview:

Electronic form

Questions About Electronic Forms	Possibilities of answers	Range scale
What's your full name?	Open answer	-
What is your child's full name?	Open answer	-
Did you have any difficulty accessing or answering the form?	Yes<br>No	0<br>1
If yes, why?	Problems with my internet<br>Difficult questions<br>Crashed the system<br>Other	-<br>-<br>-<br>-
Other...which one?	Open answer	-
As for the size of the document: Do you consider that the online question script was:	Too long and unnecessary<br>Too long but necessary<br>Should have more questions<br>Good size	0<br>1<br>2<br>3
How do you rate the ease of understanding the questions:	Difficult<br>Reasonable<br>Easy<br>Very easy	0<br>1<br>2<br>3
The online tool is:	Feasible - can be done<br>Impracticable	0<br>1
Do you recommend for other families to participate in the survey:	No<br>Maybe<br>Yes	0<br>1<br>2
If not, why not?	Open answer	-
Would you like to suggest any changes to the forms?	Yes<br>No	0<br>1
If yes, which…	Open answer	-

Voice call

Questions About the Telephone Interview	Possibilities of answers	Range scale
Did you have any difficulties?	Yes<br>No	0<br>1

If yes, why?	Very long interview<br>I have no time<br>Difficulty understanding the questions<br>Difficulty remembering answer possibilities<br>Other	-<br>-<br>-<br>-<br>-
If other… which one?	Open answer	-
Which form of assessment do you consider the best?	By online form<br>By phone call<br>I see no difference between them<br>Personally, it would be better<br>By video recording	-<br>-<br>-<br>-
Would you like to comment on anything else?	Open answer	-

Legend: EF = Electronic Form.

Table S2. Instruments applied in remote assessment of functioning and their descriptions

Instruments	ICF code	Description
YC-PEM/ PEM-CY - participation section	d210; d220; d3350; d630; d640; d649; d8800; d8803; e140; e465; e580	The The Young Children's Participation and Environment Measure for Young Chil-dren's (YC-PEM) and for Children and Youth (PEM-CY) are instruments that assess the frequency and the involvement in participation, as well as the desire for change in everyday activities that occur in the home environments, school and community, from the perspective of parents or caregivers (Coster et al, 2011, 2013, 2014; Bedell et al, 2011, Khetani et al, 2014). YC-PEM can be used in children from 0 to 5 years and PEM-CY in children and adolescents between 6 and 17 years of age, with typical development or with any type of disability, including physical, cognitive or emotional, and should be answered by parents or caregivers. For the part 'participation', in the home session there are 10 items. For each type of activity, it is questioned (1) How often the child/adolescent participated in certain situations over the last 4 months; (2) How involved the child/adolescent is when participating in 1 or 2 of the activities he/she performs most frequently; (3) If the person responsible for it would like the participation of their child/adolescent to change or not, and if so, how would he like him to change. For the part environment there are 12 questions about particular aspects of the home environment, which can be classified into two groups of four answer options, with respective scores of: 'not a problem' (4), 'usually helps' (3), 'sometimes helps/ sometimes makes harder (2), 'usually makes harder (1) as well as 'Not an issue"/'Not needed (4), Usually yes (3), Sometimes yes; sometimes not'(2) and 'Generally not'(1). The first group refers to questions about aspects that can help or make harder participation and the second group about aspects that are available or not.
YC-PEM/ PEM-CY - environment section	e240; e250	
Social Support Scale	e425; e575	It measures the social support provided to the primary caregiver of the participating child/adolescent. It has validity for the Brazilian population and high internal consistency in all its domains (Griep et al., 2005). The scale is composed of 19 items that assess, according to validation for the Brazilian population (Griep et al., 2005), three dimensions of social support: positive social interaction/affective support; emotional/information support; and material support (Griep et al., 2005). For each item, the interviewee answers, on a 5-point scale, how much he/she considers that he/she has that particular social support questioned, in the frequency of "never" (1); "rarely" (2); "sometimes" (3); "almost always" (4) and "sempre" (5) (Griep et al., 2005). The final score is obtained by the sum of the points of all items, and this will be the value used in the statistical analysis. Thus, higher scores indicate that the interviewee has greater social support.
Maternal schooling	e310	A standardized electronic questionnaire was elaborated on sociodemographic data and aspects of the experience of the pandemic, among them, the mother's schooling, which was classified on a 6-point scale, according to the level of complete education, being: 1) Incomplete Primary Education; 2) Complete Primary Education; 3) Incomplete High School; 4) Complete Higher School; 5) Incomplete Higher Education; 6) Complete High Education. This categorized value will be used in statistical analysis, and higher values indicate higher maternal schooling.
Family Income	e165	The family economic classification was performed using the Brazil Economic Classification Criterion (CCBE), according to the Brazilian Association of Research Companies - ABEP. The instrument evaluates household characteristics (presence and quantity of some household items of comfort and educational level of the family maintainer) to differentiate the population into economic classes. For each of the items evaluated, a score is defined, and the sum of points is equated with score ranges established for each class, which are in descending order A, B1, B2, C1, C2, D, E (available in: <http://www.abep.org/criterio-brasil>).

Type and Time of Social Distancing	e580	Information was collected about the social distancing to which the participant is involved about: a) type of physical social distancing (total, partial, non-isolation); b) time of distancing (for how many months the family is in physical distance, in relation to each type of distancing selected in the previous item).
IPAQ - Short Form	d460	The International Physical Activity Questionnaire short version (IPAQ-short version) was used through electronic form. The questionnaire is a valid instrument for the population of Brazilian adolescents (Guedes et al., 2005) and is the most used to assess the level of physical activity in Brazilian (Lima et al., 2018). It evaluates the frequency, duration and intensity of the activities performed by the individual, considering the week prior to the evaluation date, through eight standardized questions. Activities are classified as 'mild physical activity'; 'moderate physical activity' and 'vigorous physical activity'. The results obtained are classified as 'inactive' (sedentary), 'insufficient active A', 'insufficient active B', 'active' and 'very active' (Lima et al., 2018; Melo et al., 2009) and will be used categorically in statistical analyses, and larger categories indicate a higher level of physical activity.
Body Mass Index	b530	Body mass index (BMI) was calculated by the data filled out by mothers in the electronic forms submitted, through the arithmetic division of the individual's body weight, in kilograms, by height, in meters, squared (Sidor & Rzymski, 2020; Quadros et al., 2012). BMI indicates general body fat (CHOOI. DING. MAGKOS, 2018; WEIR, ARIF, 2021) and its classification is made in severely underweight, for values below 16.5 kg/m2, low weight below 18.5 kg/m2, normal weight between 18.5 kg/m2 and 24.9 kg/m2, overweight BMI greater than or equal (≥) to 25 kg/m2 to 29.9 kg/m2, obesity ≥ 30 kg/m2, grade I 30 to 34.9kg/m2, grade II 35 to 39.9kg/m2, grade III (severe obesity) ≥ 40 kg/m2 (OMS, 2021; WEIR, ARIF, 2021).
Sex and age	N/A	Data on the personal factors of sex and age of the child were collected through a standardized electronic form elaborated by the researchers. Age was considered a in complete years and gender was a categorical variable in males and females.
PedsQL 4.0- Parent Report<br><br>PedsQL – FIM b122	b122	The Pediatric Quality of Life Inventory (PedsQL) - version 4.0 is an instrument that assesses the functioning, disability and health of children and adolescents, being valid and reliable for the Brazilian population (Fayed et al., 2012; Klatchoian et al., 2008). The instrument can be used in children and adolescents aged 2 to 18 years, healthy or who have some type of chronic or acute health condition (https://www.pedsql.org/about_pedsql.html) and assesses the health condition of the child or adolescent based on the report of the mother. It has versions for each age group: 13-18 years, 8-12 years, 5-7 years and 2-4 years. The dimensions present in the instrument are: physical functioning (8 items), emotional functioning (5 items), social functioning (5 items) and functioning in school (5 items). Thus, the instrument has 23 items. Answer options are divided on a scale from "almost always" to "never". The final score is by converting the answers into points from 0 to 100 that are summed up and divided by the number of items answered. The higher the score, the greater the difficulties, that is, the lower the functioning. The PedsQL - FIM evaluates the functioning of the caregiver due to the health condition of their child or adolescent. It has a single version of 36 items, with aspects that involve physical capacity (6 questions), emotional aspects (5 questions), social aspects (4 questions), mental capacity (5 questions), communication (3 questions), concern (5 questions), daily activities (3 questions) and family relationships (5 questions). Participants should answer with whom often they feel the difficulty asked, being the answers between "never" and "almost always".

Table S3. Spearman correlation between remote survey feasibility and contextual factor variables.

		**Economic Level - ABEP**	**Maternal Age**	**Maternal Type of Social Distancing**	**Maternal Social Distancing Time**	**Type of Maternal Work**	**Face-to-face therapy during the pandemic**
**Feasibility**	*Rho Spearman (Correlation Coefficient)*	.049	-.290	-.007	.151	-.210	.192
	p value	.720	**.029***	.958	.261	.117	.152
***n*= 57**	*Strength of significant correlation*	-	**Weak**	-	-	-	-

Legend: Correlation strength= up to 0.19: very weak; between 0.20 and 0.39: weak; from 0.40 to 0.69: moderate; from 0.70 to 0.89: strong; and from 0.90 to 1: very strong (Cohen and Holliday 1982); *= p<0.05; ABEP = Brazilian Association of Research Companies.
